# Supplementary material for: Integrative genomic analyses identify candidate causal genes for calcific aortic valve stenosis involving tissue-specific regulation
Source: Nat Commun. 2024 Mar 18;15:2407. doi: 10.1038/s41467-024-46639-4 (PMC10944835; doi:10.1038/s41467-024-46639-4)
Supplement: Supplementary file 1 — Supplementary Information [file 41467_2024_46639_MOESM1_ESM.pdf]

SUPPLEMENTARY INFORMATION

Supplementary Note 1

Consortium members

Estonian Biobank Research Team

Andres Metspalu[1,2], Lili Milani[1], Reedik Mägi[1], Mari Nelis[1], Georgi Hudjashov[1], Tõnu Esko[1]

1. Estonian Genome Center, Institute of Genomics, University of Tartu, Tartu, Estonia

2. Institute of Cell and Molecular Biology, University of Tartu, Tartu, Estonia

Supplementary Note 2

Cohort descriptions

QUEBEC-CAVS-1

Institut universitaire de cardiologie et de pneumologie de Québec calcific aortic valve stenosis cohort phase 1 (QUEBEC-CAVS-1) is a case-control study including patients with severe aortic valve stenosis undergoing aortic valve replacement at the Institut universitaire de cardiologie et de pneumologie de Québec[1]. Only nonrheumatic cases with a tricuspid valve as determined by a cardiac surgeon upon visualization were included. The control group includes matched patients who underwent cardiac surgery mostly for coronary artery disease (>98%). The study was approved by the ethics committee of the Institut universitaire de cardiologie et de pneumologie de Québec.

QUEBEC-CAVS-2

Institut universitaire de cardiologie et de pneumologie de Québec calcific aortic valve stenosis cohort phase 2 (QUEBEC-CAVS-2) is a case-control study including patients with aortic valve stenosis undergoing aortic valve replacement at the Institut universitaire de cardiologie et de pneumologie de Québec. Nonrheumatic cases with a tricuspid or a biscuspid valve were included. The control group consists of participants of European ancestry without valvular heart disease from the CARTaGENE cohort[2]. Access to CARTaGENE for this study has been approved under project number 232977. The study was approved by the ethics committee of the Institut universitaire de cardiologie et de pneumologie de Québec.

EPIC-Norfolk

The European Prospective Investigation into Cancer and Nutrition (EPIC)-Norfolk study (DOI 10.22025/2019.10.105.00004) is a prospective population-based cohort study which recruited 25,639 men and women aged 40-79 years at baseline between 1993 and 1997 from 35

participating general practices in Norfolk, UK[3]. Individuals attended for a baseline health check including the provision of blood samples for concurrent and future analysis. Further health check visits have been conducted since the baseline visit. Participants have contributed information about their diet, lifestyle and health through questionnaires and health checks over two decades. Cases with incident calcific aortic valve stenosis were identified with International Classification of Diseases (ICD) version 9 code 395, ICD version 10 code I35 and death certificate (main cause). DNA has been extracted from all EPIC participants and stored blood has been analysed for an extensive range of classical and novel biomarkers. The Norwich Local Research Ethics Committee granted ethical approval for the study, and all participants gave written informed consent.

Estonian Biobank

The Estonian Biobank is a population-based biobank with more than 200,000 participants in the current data freeze[4]. All biobank participants have signed a broad informed consent form and information on International Classification of Diseases (ICD) codes is obtained via regular linking with the national Health Insurance Fund and other relevant databases. Calcific aortic valve stenosis cases were identified using ICD version 10 codes I35.0 and I35.2. This study and the use of data from 1,201 cases and 191,760 controls was approved by the Estonian Committee on Bioethics and Human Research (approval number 1.1-12/624).

UK Biobank

UK Biobank is a large prospective cohort of about 500,000 individuals between 40 and 69 years old recruited from 2006 to 2010 in several centers located in the United Kingdom[5]. CAVS diagnosis was established from hospital record, using the International Classification of Diseases version-10 (ICD10) and Office of Population Censuses and Surveys Classification of Interventions and Procedures (OPCS-4) coding. CAVS was defined as ICD10 code number I35.0 or I35.2. Participants with a history of rheumatic fever or rheumatic heart disease as determined by ICD10 codes I00–I02 and I05–I09 were excluded from the CAVS group. We included all other participants in the control group, except for those with OPCS-4 codes K26 (plastic repair of aortic valve) or K30.2 (revision of plastic repair of aortic valve) or a self reported diagnosis of CAVS, which were excluded from the analysis. UK Biobank received approval from the British National Health Service, North West - Haydock Research Ethics Committee (16/NW/0274). All participants of UKB provided informed consent at the baseline assessment. The present analyses were conducted under UK Biobank data application number 25205.

FinnGen

FinnGen is a large public-private partnership aiming to collect and analyse genome and health data from 500,000 Finnish participants launched in 2017[6]. The study combines genome

information with digital health record data from Finnish health registries. It includes nine Finnish biobanks, universities and university hospitals, 13 international pharmaceutical industry partners and Finnish biobank cooperative (FINBB) in a pre-competitive partnership. The project utilizes the nationwide longitudinal health register data collected since 1969 from every resident in Finland. As of June 1st, 2022, summary statistics for 3095 endpoints in 309,154 individuals are publicly available (Release 7). CAVS was defined as International Classification of Diseases (ICD) version-10 code number I35.0 or I35.2 or ICD version-9 code number 424.1 or Nordic Medico-Statistical Committee (NOMESCO) classification for medical procedures code number FMA, FMB, FMD0-1-2, FMD15, or FMD96. The Coordinating Ethics Committee of the Hospital District of Helsinki and Uusimaa (HUS) approved the FinnGen study protocol Nr HUS/990/2017. Patients and control subjects in FinnGen provided informed consent for biobank research, based on the Finnish Biobank Act.

Supplementary Fig. 1: Flowchart summarizing the study design

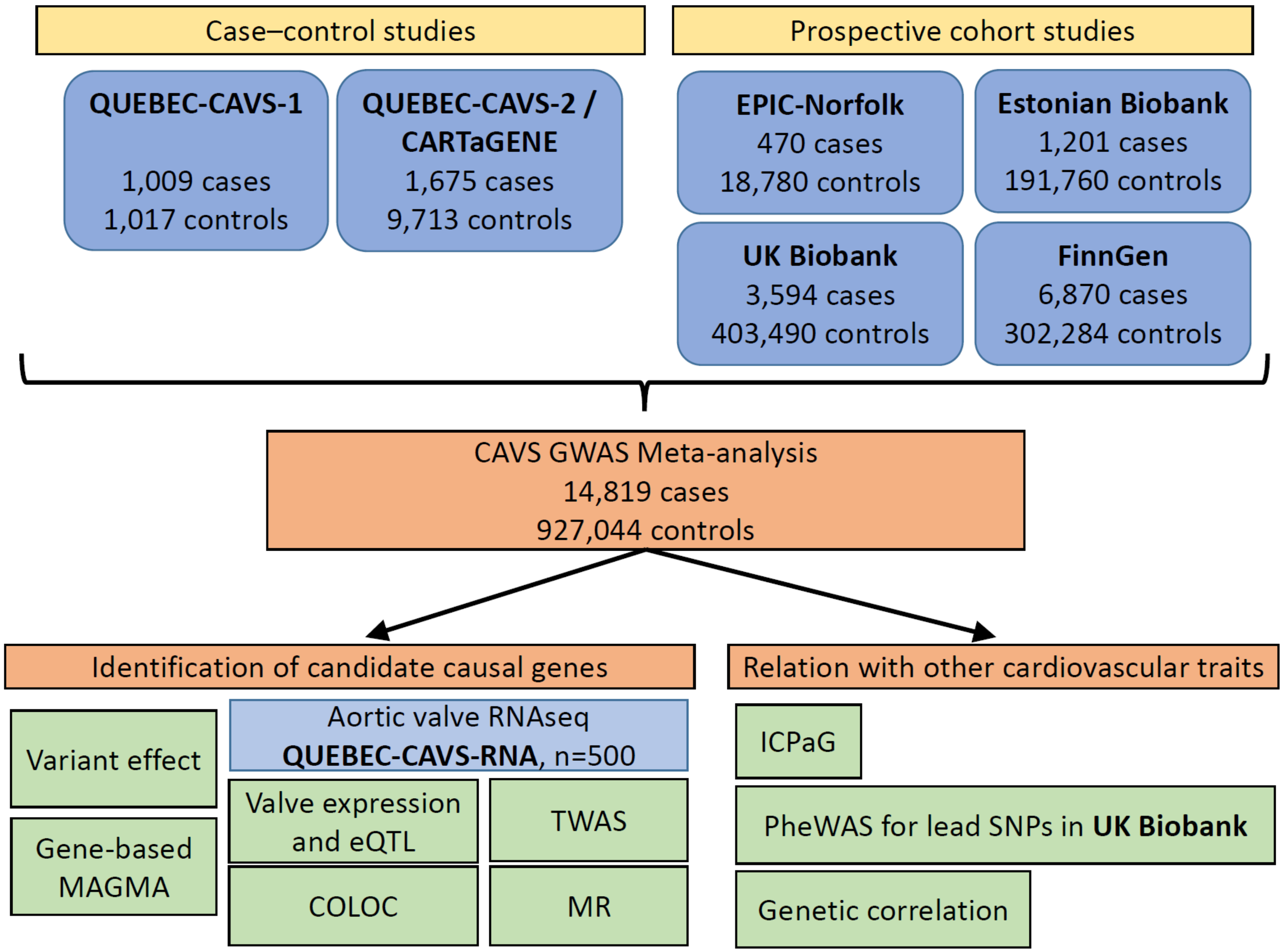

CAVS: Calcific aortic valve stenosis; GWAS: Genome-wide association study; RNAseq: RNA sequencing; eQTL: Expression quantitative trait loci; COLOC: Colocalization analyses; TWAS: Transcriptome-wide association study; MR: Mendelian randomization; PheWAS: Phenome-wide association study; ICPaG: interactive cross-phenotype analysis of GWAS database.

Supplementary Fig. 2: Quantile-quantile plot of the GWAS in each cohort

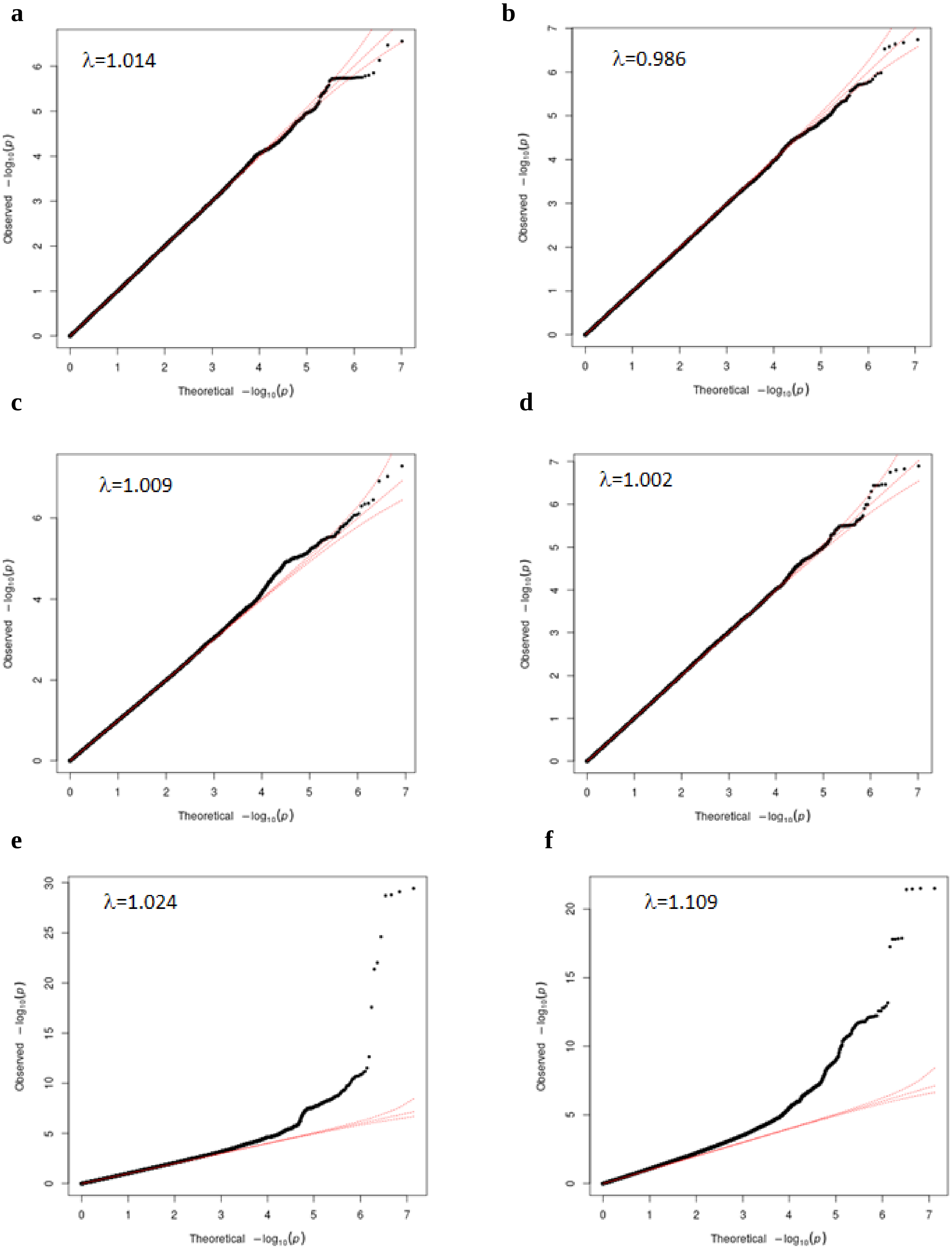

a, QUEBEC-CAVS-1. **b**, QUEBEC-CAVS-2. **c**, EPIC-Norfolk. **d**, Estonian Biobank. **e**, UK Biobank **f**, FinnGen.

Supplementary Fig. 3: Expression specificity scores of the genes located nearby the 32 genome-wide associated loci in the aortic valve and 43 tissues from the Genotype-Tissue Expression project

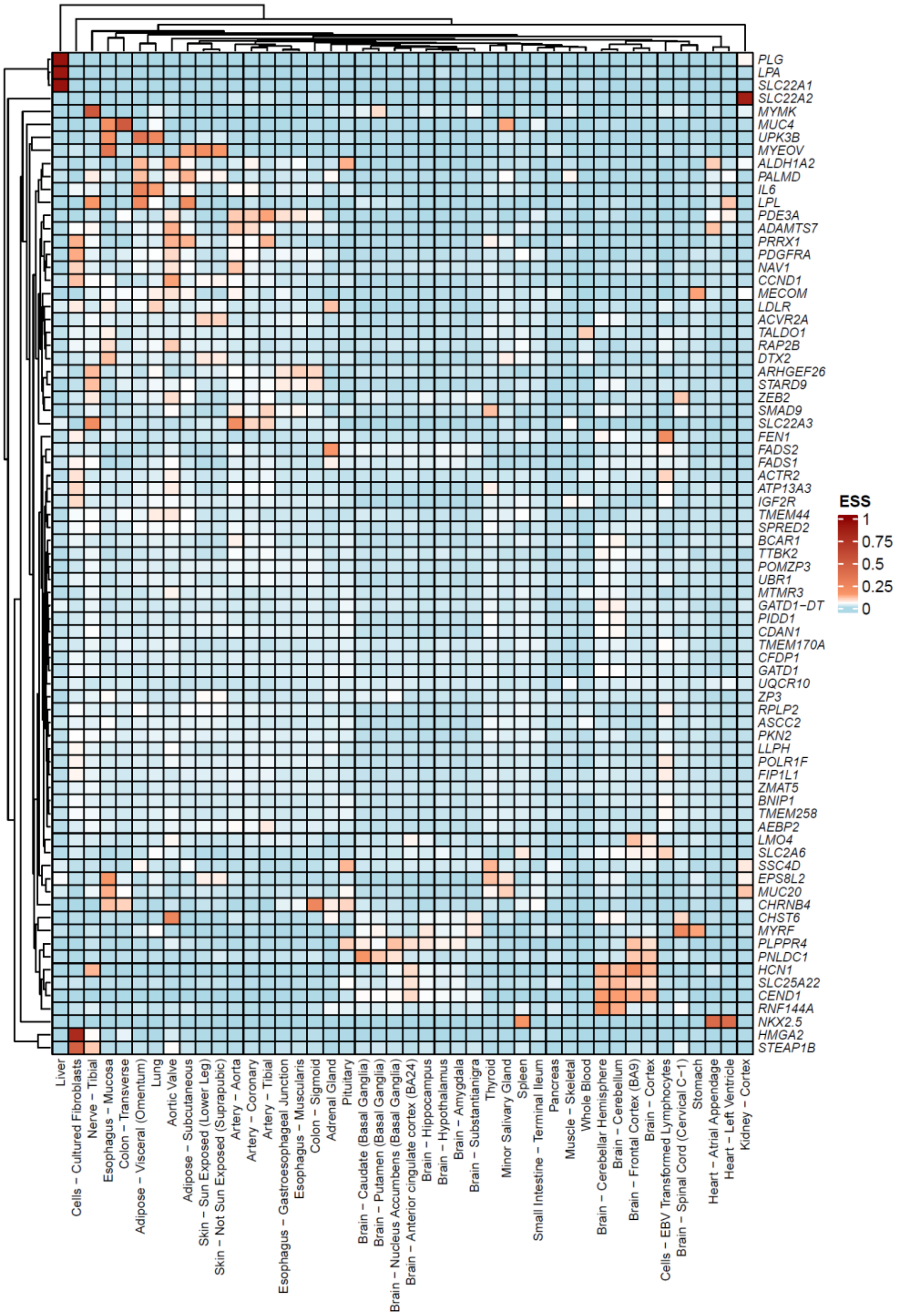

Tissues and genes are sorted according to hierarchical clustering, the aortic valve is the 8th tissue form the left. ESS: expression specificity score.

Supplementary Fig. 4: Expression quantitative trait loci at the 32 meta-analysis lead SNPs compared to all tested variants

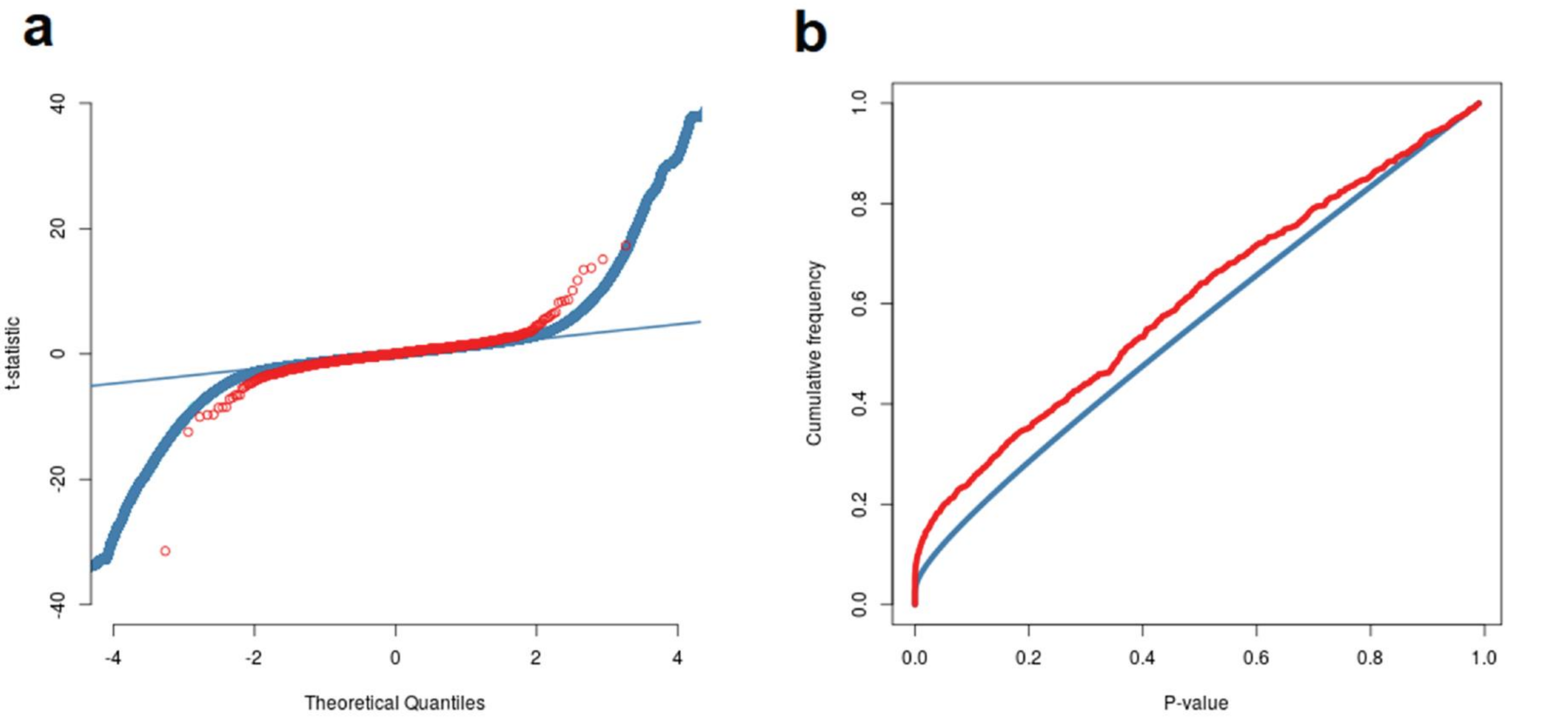

a, Quantile plot of the t-statistic for association between gene expression and genotype dosage. Red circles represent the SNP-gene pairs for the 32 meta-analysis lead SNPs (n=915). Blue circles represent all the SNP-gene pairs tested (n=182,925,823). **b**, Cumulative frequency distribution of the nominal p-values for association between gene expression and genotype dosage. The red line represents the SNP-gene pairs for the 32 meta-analysis lead SNPs (n=915). The blue line represents all the SNP-gene pairs tested (n=182,925,823).

Supplementary Fig. 5: Number of significant expression quantitative trait loci per tissue at the 32 lead meta-analysis SNPs

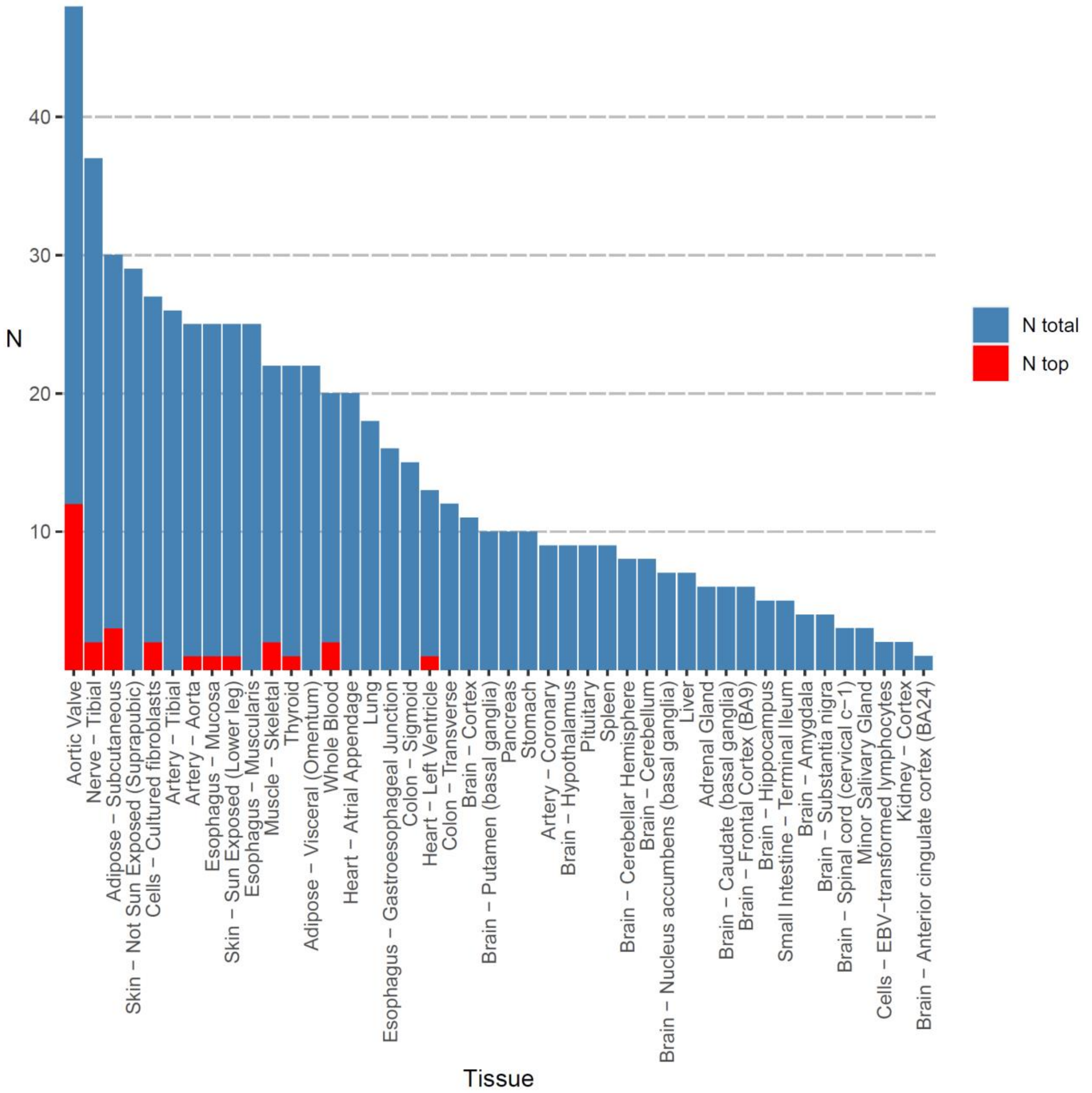

Blue bars represent the total number of significant SNP-gene pairs for each tissue. Red bars represent the number of most significant SNP-gene pairs (lowest p-value for a given locus) for each tissue.

Supplementary Fig. 6: LocusCompare plots for five candidate causal genes identified using transcriptomics in human aortic valve

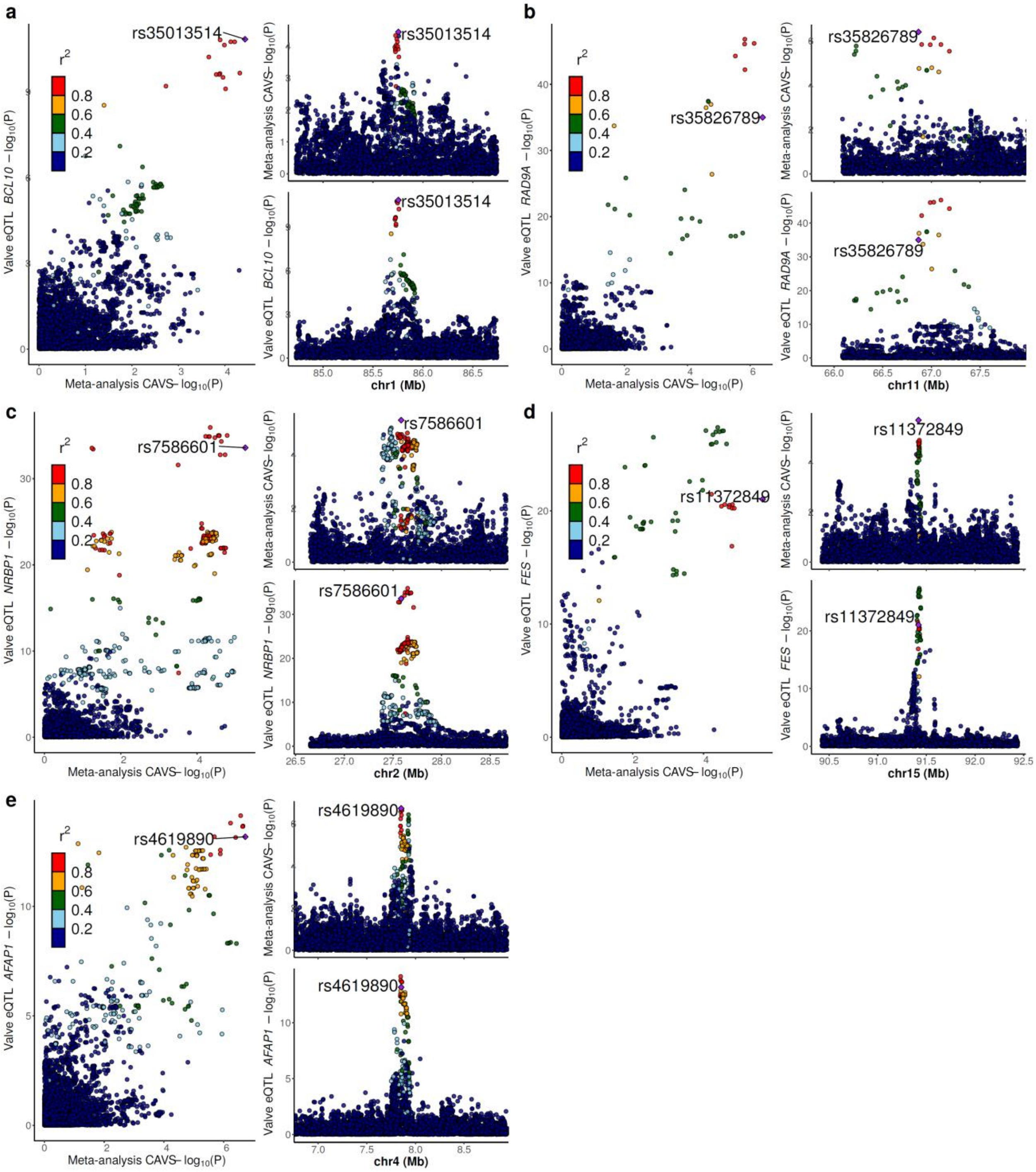

a, *BCL10.* **b,** *RAD9A.* **c,** *NRBP1.* **d,** *FES.* **e,** *AFAP1.* *P* for calcific aortic valve stenosis was obtained from the inverse-variance weighted fixed-effect GWAS meta-analysis. *P* for valve eQTL was obtained from the nominal association between genotype and normalized gene expression.

Supplementary Fig. 7: Mendelian randomization analyses for five candidate causal genes identified using transcriptomics in human aortic valve

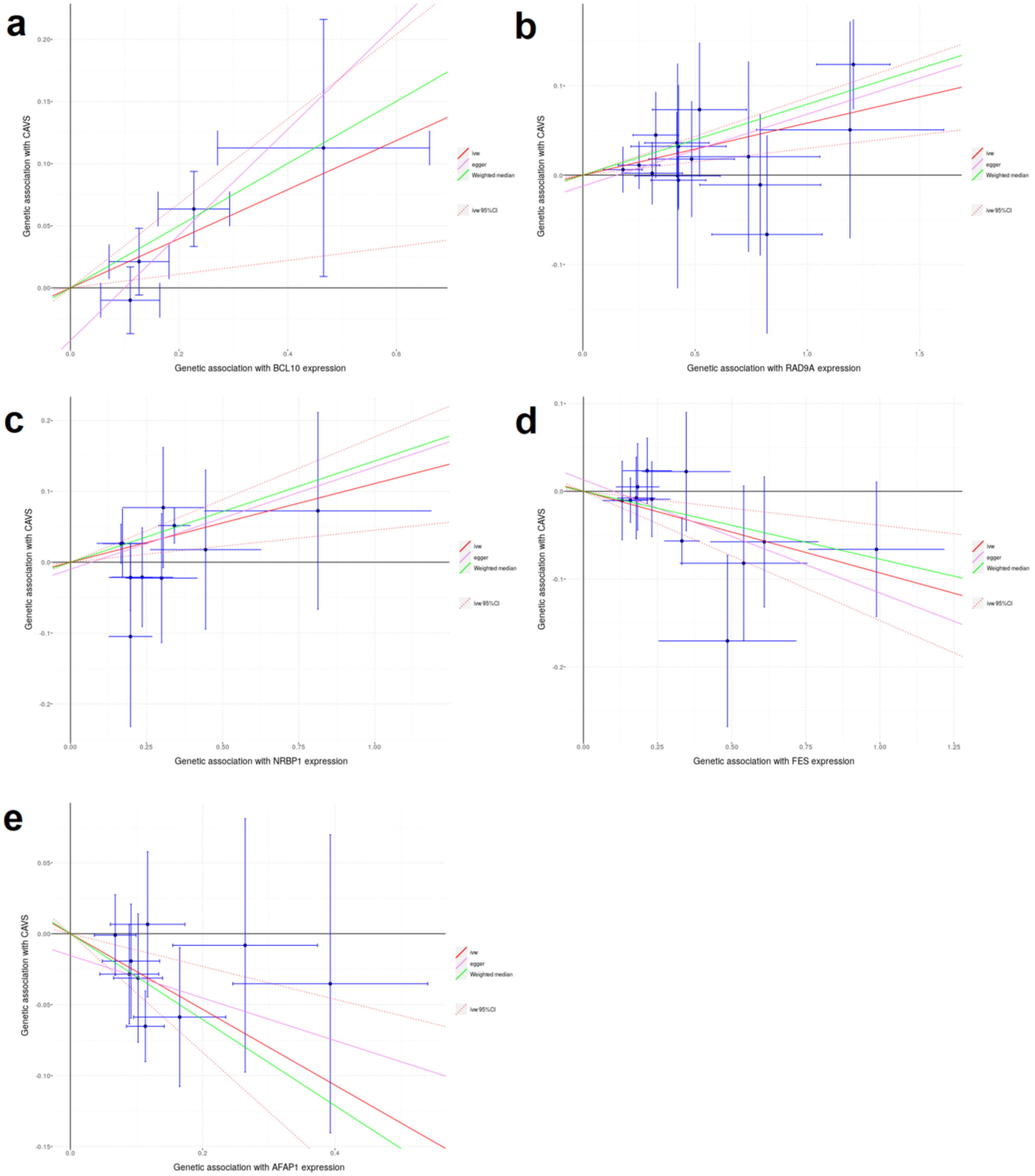

a, *BCL10*. **b,** *RAD9A*. **c,** *NRBP1*. **d,** *FES*. **e,** *AFAP1*. Data are presented as the effect and 95% confidence interval (+/- 1.96*standard error). Red line: inverse-variant weighted (IVW) MR; Dotted red lines: 95% confidence interval for IVW MR; Green line: Weighted median MR; Pink line: Egger MR.

Supplementary Fig. 8: Pathway enrichment for genes of interest.

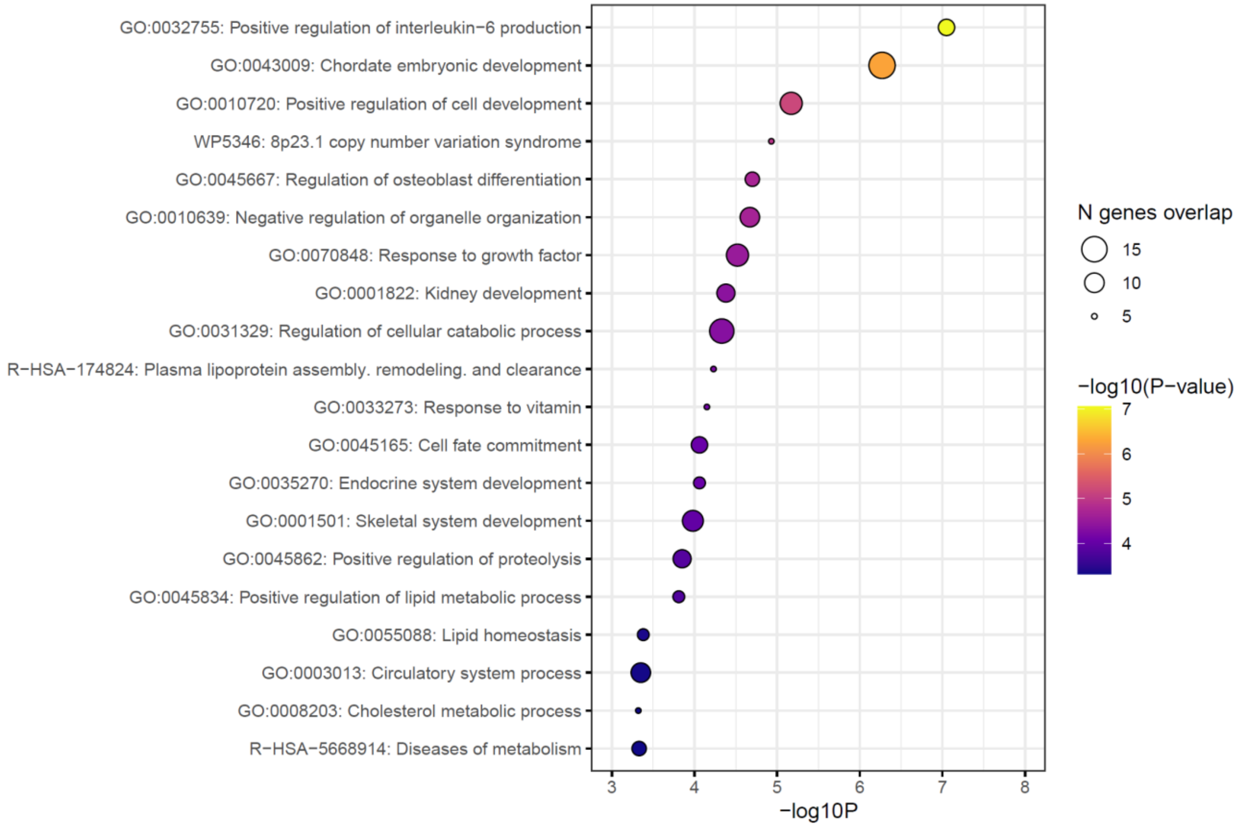

The terms were selected from Gene Ontology biological processes, KEGG pathways, Reactome gene sets, canonical pathways, and WikiPathways. The analysis was performed using Metascape with 166 genes retrieved from 173 genes of interest selected based on their significance in the MAGMA and TWAS analyses. The statistical significance of the association for each term was obtained from a hypergeometric test and is illustrated by the color (p-value). The number of overlapping genes is illustrated by the size of the bubble.

Supplementary References

1. Theriault, S. *et al.* A transcriptome-wide association study identifies PALMD as a susceptibility gene for calcific aortic valve stenosis. *Nat Commun* **9**, 988 (2018).
2. Awadalla, P. *et al.* Cohort profile of the CARTaGENE study: Quebec's population-based biobank for public health and personalized genomics. *Int J Epidemiol* **42**, 1285-99 (2013).
3. Day, N. *et al.* EPIC-Norfolk: study design and characteristics of the cohort. European Prospective Investigation of Cancer. *Br J Cancer* **80 Suppl 1**, 95-103 (1999).
4. Leitsalu, L. *et al.* Cohort Profile: Estonian Biobank of the Estonian Genome Center, University of Tartu. *Int J Epidemiol* **44**, 1137-47 (2015).
5. Bycroft, C. *et al.* The UK Biobank resource with deep phenotyping and genomic data. *Nature* **562**, 203-209 (2018).
6. Kurki, M.I. *et al.* FinnGen: Unique genetic insights from combining isolated population and national health register data. *medRxiv*, 2022.03.03.22271360 (2022).
