# Supplementary material for: Integrative genomic analyses identify candidate causal genes for calcific aortic valve stenosis involving tissue-specific regulation
Source: Nat Commun. 2024 Mar 18;15:2407. doi: 10.1038/s41467-024-46639-4 (PMC10944835; doi:10.1038/s41467-024-46639-4)
Supplement: Supplementary file 3 — Description of Additional Supplementary Files [file 41467_2024_46639_MOESM3_ESM.pdf]

Description of Additional Supplementary Files

File Name: Supplementary Data 1. Clinical characteristics of the individuals included in the six cohorts

Description: Clinical characteristics other than sex were not available for individual participants from FinnGen. BMI: body-mass index; CAD: coronary artery disease; MI: myocardial infarction.

File Name: Supplementary Data 2. Genomic loci identified in the GWAS meta-analysis for CAVS

Description: The association of each variant with calcific aortic valve stenosis was obtained from an inverse-variance weighted fixed-effect meta-analysis combining the effect per allele in the cohorts with available data. Nearby genes include protein-coding genes located within 10 kilobases of a genome-wide significant SNP or on either side of a lead intergenic SNP. RA: risk allele; NRA: non-risk allele; Dir: Direction of effect in QUEBEC-CAVS-1, QUEBEC-CAVS-2, EPIC-Norfolk, Estonian Biobank, UK Biobank and FinnGen.

File Name: Supplementary Data 3. Association between lead variants at genome-wide significant loci and CAVS in other studies

Description: The association of each variant with calcific aortic valve stenosis was obtained from an inverse-variance weighted fixed-effect meta-analysis combining the effect per allele in the cohorts with available data. For variants with no results available, we used a proxy in linkage disequilibrium (r^2>0.8) using 1000 Genomes phase 3 European as reference panel. OR: odds ratio for CAVS; *P* FDR: p-value adjusted for a false-discovery rate of 5%; Dir: Direction of effect.

1. Chen, H.Y. et al. Dyslipidemia, inflammation, calcification, and adiposity in aortic stenosis: a genome-wide study. Eur Heart J (2023).

2. Helgadottir, A. et al. Genome-wide analysis yields new loci associating with aortic valve stenosis. Nat Commun 9, 987 (2018).

File Name: Supplementary Data 4. Replication of lead variants at genome-wide significant loci reported in other studies

Description: The association of each variant with calcific aortic valve stenosis was obtained from an inverse-variance weighted fixed-effect meta-analysis combining the effect per allele in the cohorts with available data. Values in bold denote statistically significant replication. EA: effect allele; NEA: non-effect allele; *P* FDR: p-value adjusted for a false-discovery rate of 5%; P_{Het}: heterogeneity p-value (Cochran's Q-test); EAF: effect allele frequency; Dir: Direction of

effect in QUEBEC-CAVS-1, QUEBEC-CAVS-2, EPIC-Norfolk, Estonian Biobank, UK Biobank and FinnGen,

1. Chen, H.Y. et al. Dyslipidemia, inflammation, calcification, and adiposity in aortic stenosis: a genome-wide study. Eur Heart J (2023).

2. Thériault, S. et al. Genetic association analyses highlight IL6, ALPL, and NAV1 as three new susceptibility genes underlying calcific aortic valve stenosis. Circulation: Genomic and Precision Medicine 12, 431-441 (2019).

3. Small, A.M. et al. Multiancestry Genome-Wide Association Study of Aortic Stenosis Identifies Multiple Novel Loci in the Million Veteran Program. Circulation (2023).

File Name: Supplementary Data 5. Enrichment in functional annotation of variants in linkage disequilibrium with independent significant SNPs

Description: Two-sided Fisher exact test. Values in bold denote statistically significant enrichment. ncRNA: non-coding RNA.

File Name: Supplementary Data 6. Exonic variants associated with CAVS at $P<1x10^{-5}$

Description: The association of each variant with calcific aortic valve stenosis was obtained from an inverse-variance weighted fixed-effect meta-analysis combining the effect per allele in the cohorts with available data. Characters in bold indicate the top missense or loss-of-function variant in linkage disequilibrium with a lead variant at a genome-wide significant locus. EA: effect allele; NEA: non-effect allele; EAF UKB: Effect allele frequency in UK Biobank; Dir: Direction of effect in QUEBEC-CAVS-1, QUEBEC-CAVS-2, EPIC-Norfolk, Estonian Biobank, UK Biobank and FinnGen; P_{Het}: heterogeneity p-value.

File Name: Supplementary Data 7

Description: Credible set at the 32 genome-wide significant loci.

File Name: Supplementary Data 8. Loci with multiple variants identified using conditional analyses

Description: Chr: chromosome; EA: effect allele; Beta: effect size in the meta-analysis; SE: standard error in the meta-analysis; *P*: p-value in the inverse-variance weighted fixed-effect meta-analysis for calcific aortic valve stenosis; N: sample size in the meta-analysis; EAF 1000G: frequency of the effect allele in the 1000 Genomes reference sample; Beta joint: effect size in the joint analysis; SE joint: standard error in the joint analysis; *P* joint: p-value in the joint association analysis performed using COJO; LD: LD correlation with SNPs at the same locus.

File Name: Supplementary Data 9. Genes of interest selected based on their proximity with lead GWAS variants and MAGMA analyses

Description: Nearby: genes located within 10 kilobases of the lead meta-analysis SNPs or on either side of lead intergenic SNPs; MAGMA: genes significant in the MAGMA analysis at a threshold of false discovery rate <5%; *P* MAGMA: p-value for MAGMA gene association analysis; Locus: numbered genome-wide significant locus for CAVS; In RNAseq: gene available in the aortic valve RNAseq dataset and GTEx.

File Name: Supplementary Data 10. Clinical characteristics of the 500 participants included in QUEBEC-CAVS-RNA

Description: Mean ± standard deviation for continuous variables.

File Name: Supplementary Data 11. Genes of interest with high expression in the aortic valve

Description: Genes with median expression above the 90th percentile of all protein-coding genes in 500 human aortic valves (log2 transcript per million > 6.28). Characters in bold indicate the genes located within 10 kilobases of a genome-wide significant SNP or on either side of a lead intergenic SNP.

File Name: Supplementary Data 12. Expression specificity scores for CAVS genes of interest

Description: Analysis performed using gene expression in aortic valve and 43 other tissues from GTEx. Characters in bold indicate the genes with ESS>0.1 in the aortic valve and located within 10 kilobases of a genome-wide significant SNP or on either side of a lead intergenic SNP. Gene expression of *FERD3L* and *EDDM3B* was undetectable in all tissues. ESS: Expression specificity score; Max tissue: Tissue with the maximum observed ESS; ESS max: Maximum observed ESS.

File Name: Supplementary Data 13. Expression quantitative trait loci in human aortic valve for the 32 meta-analysis lead SNPs

Description: *Gene expression for ENSG00000288979, ENSG00000289268 and ENSG00000284713 was not available in GTEx v8. Characters in bold indicate an eQTL for which the strongest association was in the aortic valve and for which there was no association in other tissues (top and tissue specific). Effect RA: Normalized effect size for the CAVS risk allele; *P*: nominal p-value for the association between genotype and normalized gene expression;

Locus: numbered genome-wide significant locus for CAVS; Top eQTL: Strongest association for this lead SNP in aortic valve and 43 GTEx tissues; Tissue specific: eQTL (SNP-gene pair) only significant in the aortic valve.

File Name: Supplementary Data 14. Expression quantitative trait loci in 43 tissues from GTEx for the 32 meta-analysis lead SNPs

Description: *Proxies were used for 2 lead SNPs not found in GTEx: rs3834458 for rs174551 (r^2=0.996), rs665770 for rs682112 (r^2=0.996). Characters in bold indicate an eQTL for which the strongest association was in a relevant tissue. Effect RA: Normalized effect size for the CAVS risk allele; *P*: nominal p-value for the association between genotype and normalized gene expression; Locus: numbered genome-wide significant locus for CAVS. Top eQTL: Strongest association for this lead SNP in aortic valve and 43 GTEx tissues; Tissue specific: eQTL (SNP-gene pair) only significant in the respective tissue.

File Name: Supplementary Data 15. Candidate genes for CAVS identified using transcriptomic analyses in human aortic valves

Description: Genes in bold are candidate causal genes (PASS). Characters in red indicate results that did not reach statistical significance for the respective analysis. TWAS: transcriptome-wide association study in the aortic valve; FDR: false-discovery rate of 5%; COLOC PP4: Probability for colocalization of GWAS and expression signals; MR: Mendelian randomization for expression in the aortic valve; IVW: inverse-variant weighted approach; int: intercept; Het: Heterogeneity of the IVW instrument using Cochran's Q test; PASS: genes significant in the TWAS (*P* FDR <0.05), colocalization (PP4≥ 0.75) and MR (*P* <0.05) analyses; F-statistic provided as mean (range) of all instruments (variants).

File Name: Supplementary Data 16. eQTL according to valve morphology for the ten genes with supporting evidence from transcriptomic analyses

Description: Beta: normalized effect of the variant risk allele on gene expression; SE: standard error of the normalized effect of the variant on gene expression; *P*: nominal p-value for the association between genotype and normalized gene expression; All: analysis including all aortic valves (n=484); TAV: analysis including only tricuspid aortic valves (n=215); BAV: analysis including only bicuspid aortic valves (n=211); P_{Het}: heterogeneity p-value (Woolf's test).

File Name: Supplementary Data 17. Differentially expressed genes according to homozygous genotype at the *TWIST1* locus

Description: Differential expression analyses performed using a likelihood ratio test with adjustment for age, sex, smoking, RIN, 3' bias, alignment rate and mitochondrial gene expression

proportion. logFC: log2 fold-change, logCPM: log2 count per million, LR: likelihood ratio, FDR: False discovery rate.

File Name: Supplementary Data 18: Differential gene expression in aortic valves of individuals with severe aortic stenosis according to risk genotype at five loci

Description: HMZ: homozygous; DEG: Differentially expressed genes.

File Name: Supplementary Data 19. Enriched pathways for dysregulated genes in the aortic valve of individuals homozygous for the risk allele at the lead variant near *TWIST1*

Description: P-value for hypergeometric enrichment test. The leading independent terms identified by hierarchical clustering are in bold.

File Name: Supplementary Data 20

Description: Drugs interacting with the proteins coded by prioritized genes.

File Name: Supplementary Data 21. Enriched pathways for genes of interest selected based on MAGMA and TWAS analyses

Description: P-value for hypergeometric enrichment test. The top 20 independent terms identified by hierarchical clustering are in bold.

File Name: Supplementary Data 22. Interactive cross-phenotype analysis of GWAS

Description: Fisher`s exact test. Trait EFO: Identification number for the Experimental Factor Ontology classification used in GWAS catalog.

File Name: Supplementary Data 23. Genetic correlation analyses between CAVS and relevant traits

Description: SE: Standard error; *P*: p-value for genetic correlation as implemented in ldsc.

1. Graham, S.E. et al. The power of genetic diversity in genome-wide association studies of lipids. Nature 600, 675-679 (2021). https://csg.sph.umich.edu/willer/public/glgc-lipids2021/

2. Evangelou, E. et al. Genetic analysis of over 1 million people identifies 535 new loci associated with blood pressure traits. Nat Genet 50, 1412-1425 (2018). https://www.ebi.ac.uk/gwas/publications/30224653

3. Pirruccello, J.P. et al. Deep learning enables genetic analysis of the human thoracic aorta. Nat Genet 54, 40-51 (2022). https://www.ebi.ac.uk/gwas/publications/34837083

4. Sethi, A. et al. Calcification of the abdominal aorta is an under-appreciated cardiovascular disease risk factor in the general population. Front Cardiovasc Med 9, 1003246 (2022). https://www.ebi.ac.uk/gwas/publications/36277789

5. Wood, A.R. et al. Defining the role of common variation in the genomic and biological architecture of adult human height. Nat Genet 46, 1173-86 (2014). https://portals.broadinstitute.org/collaboration/giant/index.php/GIANT_consortium_data_files

6. Locke, A.E. et al. Genetic studies of body mass index yield new insights for obesity biology. Nature 518, 197-206 (2015). https://portals.broadinstitute.org/collaboration/giant/index.php/GIANT_consortium_data_files

7. Shungin, D. et al. New genetic loci link adipose and insulin biology to body fat distribution. Nature 518, 187-196 (2015). https://portals.broadinstitute.org/collaboration/giant/index.php/GIANT_consortium_data_files

8. Aragam, K.G. et al. Discovery and systematic characterization of risk variants and genes for coronary artery disease in over a million participants. Nat Genet 54, 1803-1815 (2022). https://www.ebi.ac.uk/gwas/publications/36474045

9. Mishra, A. et al. Stroke genetics informs drug discovery and risk prediction across ancestries. Nature 611, 115-123 (2022). https://www.ebi.ac.uk/gwas/publications/36180795

10. Sakaue, S. et al. A cross-population atlas of genetic associations for 220 human phenotypes. Nat Genet 53, 1415-1424 (2021). https://www.ebi.ac.uk/gwas/publications/34594039

11. Mahajan, A. et al. Multi-ancestry genetic study of type 2 diabetes highlights the power of diverse populations for discovery and translation. Nat Genet 54, 560-572 (2022). https://diagram-consortium.org/downloads.html

12. Stanzick, K.J. et al. Discovery and prioritization of variants and genes for kidney function in >1.2 million individuals. Nat Commun 12, 4350 (2021). https://ckdgen.imbi.uni-freiburg.de/datasets/Stanzick_2021

13. Said, S. et al. Genetic analysis of over half a million people characterises C-reactive protein loci. Nat Commun 13, 2198 (2022). https://www.ebi.ac.uk/gwas/publications/35459240

14. Morris, J.A. et al. An atlas of genetic influences on osteoporosis in humans and mice. Nat Genet 51, 258-266 (2019). https://www.ebi.ac.uk/gwas/publications/30598549

File Name: Supplementary Data 24. Genotyping and imputation methods in each cohort

Description: HWE: Hardy-Weinberg equilibrium; MAF: minor allele frequency; MAC: minor allele count.

File Name: Supplementary Data 25. List of 43 non sex-specific GTEx tissues included in the analyses

Description: Number of samples with RNAseq expression and genotypes available for each tissue.

File Name: Supplementary Data 26

Description: Description of the traits included in the phenome-wide association studies in UK Biobank.
