# Supplementary material for: Integrative genomic analyses identify candidate causal genes for calcific aortic valve stenosis involving tissue-specific regulation
Source: Nat Commun. 2024 Mar 18;15:2407. doi: 10.1038/s41467-024-46639-4 (PMC10944835; doi:10.1038/s41467-024-46639-4)
Supplement: Supplementary file 5 — Reporting Summary [file 41467_2024_46639_MOESM5_ESM.pdf]

nature portfolio

Corresponding author(s): Sébastien Thériault

Last updated by author(s): Feb 2, 2024

Reporting Summary

Nature Portfolio wishes to improve the reproducibility of the work that we publish. This form provides structure for consistency and transparency in reporting. For further information on Nature Portfolio policies, see our Editorial Policies and the Editorial Policy Checklist.

Statistics

For all statistical analyses, confirm that the following items are present in the figure legend, table legend, main text, or Methods section.

n/a	Confirmed	
☐	☒	The exact sample size (*n*) for each experimental group/condition, given as a discrete number and unit of measurement
☐	☒	A statement on whether measurements were taken from distinct samples or whether the same sample was measured repeatedly
☐	☒	The statistical test(s) used AND whether they are one- or two-sided<br>*Only common tests should be described solely by name; describe more complex techniques in the Methods section.*
☐	☒	A description of all covariates tested
☐	☒	A description of any assumptions or corrections, such as tests of normality and adjustment for multiple comparisons
☐	☒	A full description of the statistical parameters including central tendency (e.g. means) or other basic estimates (e.g. regression coefficient) AND variation (e.g. standard deviation) or associated estimates of uncertainty (e.g. confidence intervals)
☐	☒	For null hypothesis testing, the test statistic (e.g. *F*, *t*, *r*) with confidence intervals, effect sizes, degrees of freedom and *P* value noted<br>*Give P values as exact values whenever suitable.*
☒	☐	For Bayesian analysis, information on the choice of priors and Markov chain Monte Carlo settings
☒	☐	For hierarchical and complex designs, identification of the appropriate level for tests and full reporting of outcomes
☐	☒	Estimates of effect sizes (e.g. Cohen's *d*, Pearson's *r*), indicating how they were calculated

Our web collection on statistics for biologists contains articles on many of the points above.

Software and code

Policy information about availability of computer code

Data collection	No software was used for data collection.
Data analysis	Analyses were performed using R version 3.5.1 and the following packages: LocusCompareR v1.0.0, coloc v3.2.1, MendelianRandomization v0.4.3, edgeR v3.24.3, EnvStats v2.7.0, qqman v0.1.4, ggpubr v0.2.4, ggplot2 v3.2.1, statmod v1.4.35, dplyr v1.0.2, data.table v1.13.6, reshape2 v1.4.3.<br>The SNPTEST v2.5.4, SAIGE v0.39, regenie v2.0.2, METAL, ANNOVAR, CAVIAR, gcta v1.92.3beta3, MAGMA v1.08, FastQC v0.11.5, MultiQC v1.10, STAR v2.5.1b, QualiMap v2.2.1, RNA-SeQC 2, QTLtools v1.1, tensorQTL v1.0.7, MetaXcan v0.7.4, PredictDB, plink v1.9, Metascape, iCPAGdb and ldsc v1.0.0 softwares were used (references in the manuscript).

For manuscripts utilizing custom algorithms or software that are central to the research but not yet described in published literature, software must be made available to editors and reviewers. We strongly encourage code deposition in a community repository (e.g. GitHub). See the Nature Portfolio guidelines for submitting code & software for further information.

Data

Policy information about availability of data

All manuscripts must include a data availability statement. This statement should provide the following information, where applicable:

- Accession codes, unique identifiers, or web links for publicly available datasets
- A description of any restrictions on data availability
- For clinical datasets or third party data, please ensure that the statement adheres to our policy

Summary statistics of the meta-analysis generated in this study have been deposited in the NHGRI-EBI GWAS catalog under accession code XXX (https://www.ebi.ac.uk/gwas/). The RNA sequencing data from the 500 human aortic valves generated in this study have been deposited in dbGaP under accession code XXX (https://www.ncbi.nlm.nih.gov/gap/). The data are available under restricted access in accordance with the institutional ethics approval. Access can be obtained for research related to cardiovascular diseases by not-for-profit organizations providing a local institutional review board approval and a letter of collaboration with the study investigators. Requests can be made to the corresponding author who will respond within two weeks. The GWAS summary statistics from FinnGen are available here: https://www.finngen.fi/en/access_results. The summary statistics of the GWAS meta-analysis for CAVS by Chen et al.15 used in this study are available here: https://zenodo.org/records/7829401. The GWAS summary statistics for CAVS from the deCODE cohort used in this study are available here: https://www.decode.com/summarydata/. The GTEx project v8 data used in this study are available here: https://gtexportal.org/home/datasets. Individual-level and genotype data from QUEBEC-CAVS-1 and QUEBEC-CAVS-2 are available under restricted access for legal and ethical reasons. Requests can be made to the corresponding author who will respond within two weeks. Access to individual data from CARTaGENE (https://cartagene.qc.ca/), EPIC-Norfolk (https://www.epic-norfolk.org.uk/), Estonian Biobank (https://genomics.ut.ee/en/content/estonian-biobank), UK Biobank (https://www.ukbiobank.ac.uk/) and FinnGen (https://www.finngen.fi/) is available for registered researchers following the respective application process. Further information on data access is available from the study websites. The drug interaction data used in this study are available from the drug-gene interaction database (www.dgidb.org).

Research involving human participants, their data, or biological material

Policy information about studies with human participants or human data. See also policy information about sex, gender (identity/presentation), and sexual orientation and race, ethnicity and racism.

Reporting on sex and gender	Only individuals for which the genetically identified sex (based on the X and Y chromosome variants) matches the self-reported sex were included in the analysis, as a commonly used quality control procedure for genetic analyses. All the analyses were adjusted for sex (GWAS, eQTL, differential expression). We did not perform sex-stratified analyses, due to power limitations.
Reporting on race, ethnicity, or other socially relevant groupings	Only individuals of European ancestry confirmed by principal component analysis from the genotype data were included, since the sample size for other ancestries was too low.
Population characteristics	A total of six cohorts were included in the genome-wide association analyses for calcific aortic valve stenosis: two case-control cohorts and four population-based cohorts. In total, there were 14,819 cases (61% men, mean age of 67 years) and 927,044 controls (43% men, mean age of 55 years). For the RNA sequencing analyses in aortic valve tissues, there were 500 participants who underwent aortic valve replacement or heart transplant (65% men, mean age of 63 years).
Recruitment	In the two case-control cohorts (QUEBEC-CAVS-1 and QUEBEC-CAVS-2) and the aortic valve tissues cohort (QUEBEC-CAVS-RNA), participants were recruited in the hospital. All patients undergoing heart surgery are approached to be included in the institutional biobank. The positive response rate is high, therefore we do not expect significant biases. The aortic valve tissues were selected to represent both men and women as well as different valve morphology. The individuals in these cohorts mostly suffer from severe aortic valve stenosis requiring surgery (as opposed to milder disease severity). In the four population-based cohorts (European Prospective Investigation into Cancer and Nutrition [EPIC] – Norfolk, Estonian Biobank [EstBB], UK Biobank and FinnGen), individuals meeting the age and location inclusion criteria were invited to participate.
Ethics oversight	The QUEBEC-CAVS study was approved by the ethics committee of the Institut universitaire de cardiologie et de pneumologie de Québec. The Norwich Local Research Ethics Committee granted ethical approval for the analysis in the European Prospective Investigation into Cancer and Nutrition [EPIC]-Norfolk study. The analysis in the Estonian Biobank [EstBB] was approved by the Estonian Committee on Bioethics and Human Research. UK Biobank received approval from the British National Health Service, North West - Haydock Research Ethics Committee. The Coordinating Ethics Committee of the Hospital District of Helsinki and Uusimaa (HUS) approved the FinnGen study protocol. Informed consent was obtained for all participants.

Note that full information on the approval of the study protocol must also be provided in the manuscript.

Field-specific reporting

Please select the one below that is the best fit for your research. If you are not sure, read the appropriate sections before making your selection.

☒ Life sciences ☐ Behavioural & social sciences ☐ Ecological, evolutionary & environmental sciences

For a reference copy of the document with all sections, see nature.com/documents/nr-reporting-summary-flat.pdf

Life sciences study design

All studies must disclose on these points even when the disclosure is negative.

Sample size	All available data from the studied cohorts and databases were used to maximize power. The RNA sequencing experiment in human aortic valve is the largest dataset of gene expression in this tissue. It is comparable in size to other large-scale transcriptomic datasets (e.g., GTEx, for which no data is available for the aortic valve).
Data exclusions	No data were excluded, except for pre-established quality control steps on the genomic data.
Replication	Data from six different cohorts were included in the GWAS meta-analysis. Heterogeneity measures are provided to document the consistency of the associations. We provide the association of the 32 lead variants identified with CAVS from two other studies (a previous GWAS meta-analysis and an independent cohort). The 32 lead variants at the genome-wide associated loci had a concordant direction of effect in the previous meta-analysis. Thirty showed nominal association with CAVS ($P<0.05$), of which 29 remained significant when using a threshold of false discovery rate <5%. In the independent cohort (n=2,457 CAVS cases and 349,342 controls), 28 out of the 32 lead variants had the same direction of effect. The use of transcriptomic data from human aortic valve tissues in combination with the genetic association results also supports the role of the genes identified.
Randomization	Not relevant to the current study, as there was no intervention. Diagnosis of calcific aortic valve stenosis was based on medical records.
Blinding	Not relevant to the current study, as there was no intervention.

Reporting for specific materials, systems and methods

We require information from authors about some types of materials, experimental systems and methods used in many studies. Here, indicate whether each material, system or method listed is relevant to your study. If you are not sure if a list item applies to your research, read the appropriate section before selecting a response.

Materials & experimental systems

n/a	Involved in the study
☒	☐ Antibodies
☒	☐ Eukaryotic cell lines
☒	☐ Palaeontology and archaeology
☒	☐ Animals and other organisms
☒	☐ Clinical data
☒	☐ Dual use research of concern
☒	☐ Plants

Methods

n/a	Involved in the study
☒	☐ ChIP-seq
☒	☐ Flow cytometry
☒	☐ MRI-based neuroimaging
